# Steroids and Olfactory Training for Postviral Olfactory Dysfunction: A Systematic Review

**DOI:** 10.3389/fnins.2021.708510

**Published:** 2021-08-12

**Authors:** Fan Yuan, Tianhao Huang, Yongxiang Wei, Dawei Wu

**Affiliations:** ^1^Department of Otolaryngology, Smell and Taste Center, Beijing Anzhen Hospital, Capital Medical University, Beijing, China; ^2^Department of Otorhinolaryngology Head and Neck Surgery, Capital Institute of Pediatrics, Beijing, China

**Keywords:** olfactory training, steroid, postviral olfactory dysfunction, systematic review, olfactory dysfunction

## Abstract

**Background:** Postviral olfactory dysfunction (PVOD) is a clinical challenge due to limited therapeutic options and poor prognosis. Both steroids and olfactory training have been proved to be effective for olfactory dysfunction with varied etiologies. We sought to perform a systematic review to summarize the evidence of steroids or olfactory training for patients with PVOD.

**Methods:** A systematic literature review using PubMed, Embase, Cochrane Library, and Web of Science was conducted to identify studies assessing olfactory change in patients with PVOD receiving steroid or olfactory training.

**Results:** Of the initial 273 abstracts reviewed, 20 articles with data from 2,415 patients with PVOD were included. Treatments including topical steroids, systemic steroids, classical olfactory training (COT), modified olfactory training (MOT), and olfactory training with steroid were analyzed. Both psychophysical olfactory testing and subjective symptom scores were utilized to assess the olfactory function. The routine use of nasal steroid spray alone during the management of PVOD seems to have no positive effect on olfactory dysfunction. Direct injection of steroid or nasal steroid spray into the olfactory cleft significantly improved the olfactory function in patients with PVOD. Olfactory improvement is greater than that of the natural course of the disease with short-term COT. Patients with PVOD would benefit more from long-term COT (>12 weeks). Treatment duration, various odorants, olfactory training devices, changing the types of odors periodically, different molecular odorants, and different concentrations of odorants tended to increase the efficiency of MOT. Clinically significant improvement after olfactory training was defined as an increase of threshold, discrimination, and identification (TDI) score ≥6. From week 24 to week 36, both COT and MOT groups reached the maximum therapeutic effect regarding the number of participants achieving clinically significant improvement. A combination of local or oral steroids with olfactory training is more efficient than COT only.

**Conclusion:** Olfactory function in patients with PVOD was effectively improved through direct steroid administration in the olfactory cleft, COT, or modification of COT. The addition of topical steroids to COT therapy showed a tendency for greater olfactory improvement in patients with PVOD.

## Introduction

Olfaction, together with vision, hearing, taste, and touch, constitutes the special sensory function of human beings, which has the effects of discriminating odors, increasing appetite, and warning, and is the primary tool for human understanding and cognition of the outside world like vision and hearing. There are numerous etiologies of olfactory dysfunction. Among them, sinonasal disease (30%), upper respiratory tract infection (URTI) (25%), and head trauma (14%) are the most common causes of olfactory dysfunction, followed by idiopathic causes (12%), congenital anosmia (3%), or others (16%) (Temmel et al., 2002; Hummel et al., 2017a; Schäfer et al., 2021).

Postviral olfactory dysfunction (PVOD) occurs after a common or epidemic URTI and is generally considered to be caused by a viral infection (Temmel et al., 2002; Seiden, 2004; Welge-Lüssen and Wolfensberger, 2006). Olfactory dysfunction has been noted as a common symptom in 18 to 22% of cases attributed to viral etiology (Tian et al., 2020). At present, the novel coronavirus disease 2019 (COVID-19) is caused by severe acute respiratory syndrome coronavirus 2 (SARS-CoV-2) infection and proves to have smell and taste loss (Printza et al., 2021). This pandemic has regained interest in PVOD and the related treatment.

A variety of drugs were reported in the literature for the treatment of PVOD, which were confirmed to affect including corticosteroids, vitamin A, *Ginkgo biloba* extract, and sodium citrate (Seo et al., 2009; Jiang et al., 2015; Hummel et al., 2017b; Whitcroft et al., 2017). Both oral and intranasal corticosteroids significantly improve olfactory function in patients with olfactory dysfunction with varied etiologies (Schriever et al., 2012; Kim et al., 2017; Nguyen and Patel, 2018). Meanwhile, olfactory training is currently the non-medical treatment supported by level 1A evidence which was proved to have a significant improvement on olfactory function in patients with olfactory disorders (Hummel et al., 2009; Patel, 2017). These treatments gained widespread acceptance and were included in treatment guidelines for PVOD (Sorokowska et al., 2017; Kattar et al., 2020; Addison et al., 2021). The objective of this systematic review was to summarize the current evidence of steroids or olfactory training in PVOD, especially a combination of steroids and olfactory training.

## Materials and Methods

### Literature Search Strategy

**Figure 1:**
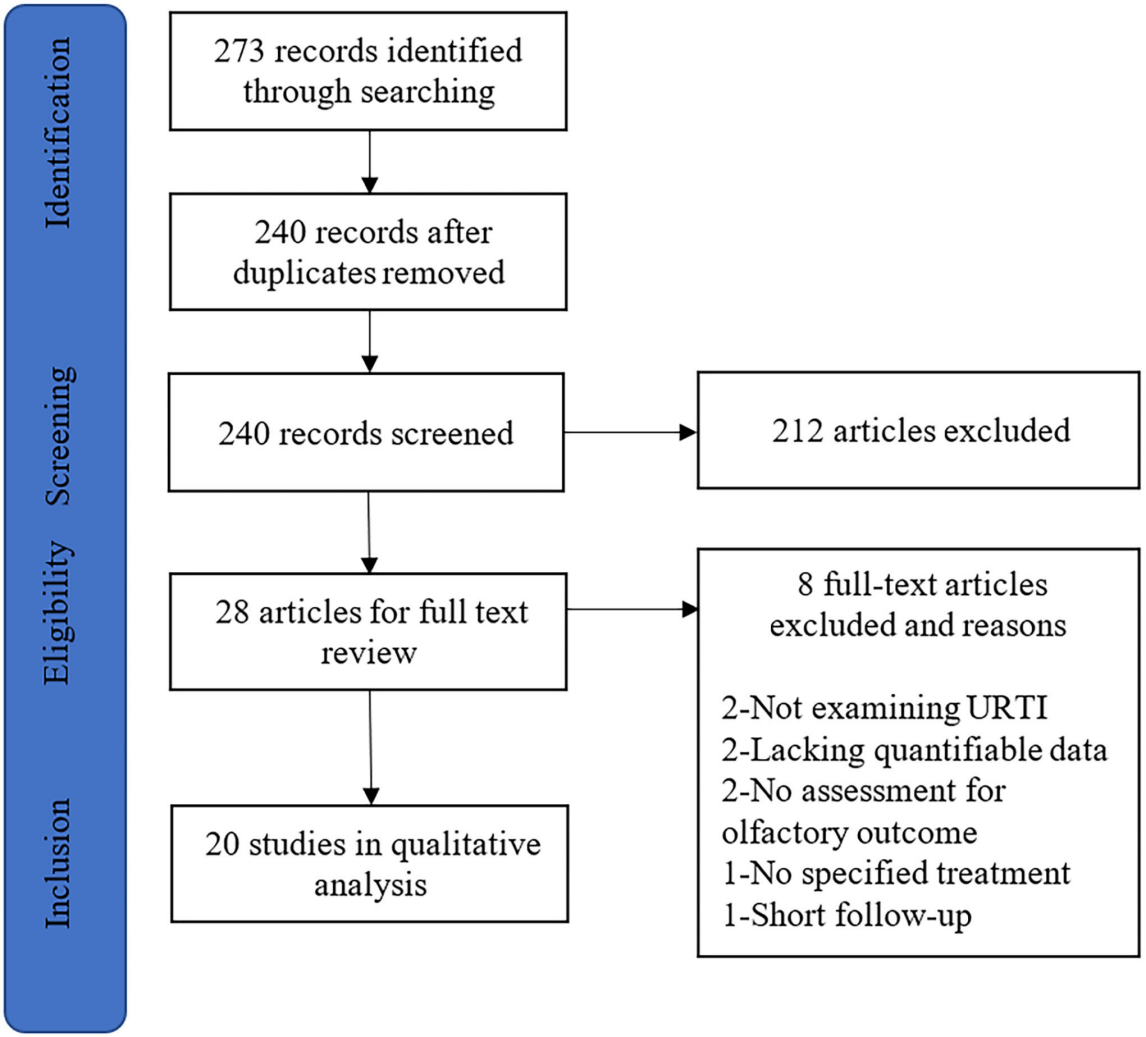
The article selection process for systematic review. URTI, upper respiratory tract infection.

A systematic search of PubMed, Cochrane Library, Embase, Google Scholar, and Web of Science databases was conducted on October 1, 2020. Two investigators (F.Y. and D.W.) independently reviewed the titles and abstracts of all studies, making the articles meet our criteria for inclusion. A combination of the following search algorithm was used in this review: postviral olfactory dysfunction, postviral anosmia, post-infectious olfactory dysfunction, post-infectious olfactory loss, or postviral olfactory disorder and olfactory therapy, olfactory training, smell training, smell therapy, steroid, systemic steroid, topical steroid, or local steroid. The search strategy is illustrated in Figure 1. This systematic review was conducted according to the Preferred Reporting Items for Systematic Reviews and Meta-analyses (PRISMA) statement (Moher et al., 2009). The systematic search was not restricted to any specific study or publication type to ensure a thorough evaluation of the literature.

### Inclusion and Exclusion Criteria

Studies exploring the effects of steroids, olfactory training, or both interventions on olfaction in patients with PVOD were included. Changes in olfactory scores or rates of patients with treatment response should be reported and abstracts containing subjects with postviral olfactory dysfunction and other etiologies of olfactory dysfunction were also included. Exclusion criteria included non-English language and patient populations composed exclusively of those with olfactory dysfunction secondary to etiologies other than viral infection (e.g., idiopathic, trauma, and chronic rhinosinusitis). Studies without a defined intervention were excluded. In addition, case reports, letters to the editor, abstracts, and book chapters were not included.

### Data Extraction and Analysis

Two reviewers (F.Y. and D.W.) each manually extracted data from studies meeting inclusion criteria. Extracted data included descriptive baseline characteristics, intervention data (regimen and duration), follow-up, and olfactory outcomes. Summary tables were developed after the extraction of articles. The quality of each article was assessed by the Oxford Center for Evidence-Based Medicine Levels of Evidence categorization (Burns et al., 2011).

## Results

### Study Characteristics

Our search identified 273 studies that met the inclusion criteria through the initial literature (Figure 1). After the removal of duplicates and abstract screening, 245 articles were excluded. Of these remaining 28 studies, 8 studies were excluded due to the following reasons: not examining postviral olfactory dysfunction (*n* = 2), lacking quantifiable data (*n* = 2), no assessment for the olfactory outcome (*n* = 2), no specified therapy (*n* = 1), and short follow-up (*n* = 1). Finally, 20 articles were included in the systematic review.

### Trial Characteristics

A total of 2,415 patients with olfactory dysfunction were included and 60.1% (1,451) of patients were PVOD. The severity of olfactory dysfunction ranged from hyposmia to anosmia. Olfactory outcomes assessed in 20 studies utilized Sniffin' Sticks, butanol threshold testing (BTT), Cross-Cultural Smell Identification Test (CCSIT), Connecticut Chemosensory Clinical Research Center test (CCCRC), Toyota & Takagi olfactometer (T&T), University of Pennsylvania Smell Identification Test (UPSIT), phenyl ethyl alcohol (PEA) threshold testing, and visual analog scale (VAS). The length of follow-up varied from half a month to 12 months. Treatment measures included topical steroids, systemic steroids, classical olfactory training (COT), and modified olfactory training (MOT). COT was defined as the regimen firstly described by Hummel et al., which involved twice-daily exposure to a set of four odors, including rose, eucalyptus, lemon, and cloves, from media such as brown jars or markers. To define the treatment response, olfactory improvement was considered with a decrease in recognition threshold score (T&T) >1 point, an increase in BTT score ≥3, an increase in CCSIT score ≥3, an increase in Sniffin' Sticks score ≥5.5 or ≥6, and an increase in UPSIT score ≥5. Minimal clinically important difference (MCID) was defined as a ≥6 increase in TDI scores (Jaeschke et al., 1989). Seventeen out of the 20 included articles had control groups and all articles were statistically analyzed.

### Systemic and Topical Steroids

Four trials used oral steroids with other control treatments and two trials used topical steroids alone (Table 1) (Heilmann et al., 2004; Fukazawa, 2005; Seo et al., 2009; Fleiner and Goktas, 2011; Schriever et al., 2012; Vaira et al., 2021). Of these trials following systemic steroids, two studies evaluated patients with PVOD and demonstrated a significant improvement of olfactory score and recovery rate after systemic steroids therapy alone (Heilmann et al., 2004; Schriever et al., 2012). The mean TDI score in patients with PVOD receiving oral steroids changed from 15.2 to 19.6 (Heilmann et al., 2004). Similarly, another study by Schriever et al. showed the TDI scores improved from 14.39 to 18.86 in patients with PVOD receiving oral steroids and 29.6% of patients with PVOD reported an increase of more than 6 points of the TDI score (Schriever et al., 2012).

**Table 1:** Summary of topical and systemic steroid studies included in the systematic review.

**References**	**Design**	**Patients**	**Intervention**	**Treatment details**	**The time of initiation of therapy**	**Follow-up**	**Improvement**	**Conclusion**	**Level of evidence**
Heilmann et al. (2004)	Retrospective, non-randomized, parallel-group case series	Systemic steroids group (*n* = 12) vs. Topical steroids group (*n* = 10) All PVOD	(1) Oral prednisolone (2) Mometasone spray	(1) Prednisolone 40 mg × 21-day taper(2) Mometasone 0.1 mg/nasal cavity × 1–3 months	5.7 years after clinical onset	21–330 days	Sniffin' Sticks Systemic steroids group Threshold: 2.7 to 3.8 Discrimination: 7 to 8.9 Identification: 5.4 to 6.9 The mean TDI score: 15.2 to 19.6 Topical steroids group Threshold: 4.2 to 4.5 Discrimination: 8.3 to 8.7 Identification: 7.4 to 6.4 The mean TDI score: 20 to 19.6	Topical application of steroids appears to have no positive effect on olfactory dysfunction, apart from a tendency of improved odor threshold and discrimination. Systemic steroids lead to improvement of olfactory function.No statistical difference between systemic and topical steroids	4
Schriever et al. (2012)	Retrospective case series	Systemic steroids PVOD: 27/425 Sinonasal (*n* = 221) Idiopathic (*n* = 157) Other causes (*n* = 20)	Oral methylprednisolone (all)	Methylprednisolone 40 mg daily with taper	5.6 years after clinical onset	2 weeks	An increase in Sniffin' Sticks score ≥6 PVOD etiology: 29.6% The TDI scores improved from 14.39 to 18.86 (*p* = 0.003)	PVOD patients exhibited clinically significant improvement in TDI after treatment with systemic steroids.	4
Fukazawa (2005)	Prospective, non-controlled case series	All PVOD(*n* = 133)	Dexamethasone or betamethasone (all)	Dexamethasone 5 mg or betamethasone 5 mg injections into olfactory cleft every 2 weeks for 16–20 weeks	–	6 months	T&T score improvement at least one point in odor recognition threshold: PVOD: 49.6% The mean points of VAS improved from 10.2 at pretreatment to 39.5 after the treatment.	Injecting the steroid into the nasal mucosa near the olfactory cleft demonstrated to improve the points of T&T olfactometry.	4
Fleiner and Goktas (2011)	Prospective case series	PVOD: 8/18	Beclomethasone spray (all)	Beclomethasone spray 250 μg directed to the olfactory cleft twice daily	2.3 months after clinical onset	4 weeks	An increase in Sniffin' Sticks score ≥6: PVOD: 25% The mean TDI score: 13.5 to 18.5	The direct application of BDP-spray to the olfactory cleft attained superior therapeutic effects than a usually applied mometasone spray.	4
Seo et al. (2009)	Randomized, non-blinded, parallel group	No *Ginkgo biloba* group (*n* = 28) vs. With *G. biloba* group (*n* = 43) All PVOD	(1) Oral prednisolone + mometasone spray (2) Oral prednisolone + mometasone spray + *G. biloba*	(1) Prednisolone 30 mg daily with taper(2) Mometasone two puffs/nasalcavity twice daily(3) *G. biloba* 80 mg three times daily	3.5 months after clinical onset	4 weeks	An increase in BTT score ≥3: No *G. biloba* group: 32% With *G. biloba* group: 37% Mean odor threshold: 4.8 to 6.7 An increase in CCSIT score ≥3: No *G. biloba* group: 14% With *G. biloba* group: 33% Mean odor identification: 3.8 to 5.3	Combination therapy with oral prednisolone and *G. biloba* did not show significantly better efficacy than monotherapy with oral prednisolone.*G. biloba* might help improve odor identification.	2B
Vaira et al. (2021)	Prospective, randomized controlled trial	Control group (*n* = 9) vs. Systemic prednisone and nasal irrigation group (*n* = 9) All PVOD	(1) No treatment (2) Systemic prednisone and nasal irrigation with betamethasone, ambroxol, and rinazine	Systemic cortisone therapy with prednisone, starting with 1 mg/kg/day and tapering the dose for 15 days and nasal irrigation with betamethasone, ambroxol, a mucolytic, and rinazine, a decongestant, for 15 days.	1 month after clinical onset	40 days	The threshold and identification test scores were finally converted into the CCCRC composite score which allows classifying the olfactory function of patients in normal (score 90 and 100), mild hyposmia (score 70 and 80), moderate hyposmia (score 50 and 60), severe hyposmia (score between 20 and 40), and anosmia (score 0 and 10) (27–30).	The mix of drugs including steroids could represent a useful specific therapy to reduce the prevalence of this long-term morbidity.	1B
Stenner et al. (2008)	Retrospective case series	Topical steroids group vs. Topical steroids + topical antibiotics group URTI: 31/89	(1) Oral betamethasone + budesonide spray (2) Oral betamethasone + budesonide spray + neomycin spray	All patients treated with 20 days oral beclomethasone(1) Betamethasone 3.0 mg daily with taper(2) Budesonide spray 1.5 mg twice daily(3) Neomycin spray 7.5 mg twice daily	4.6 years after clinical onset	12 weeks	The mean TDI of 27% of patients improved from 15.5 raised to 18.7 after the experiment.	No change with topical treatment of both steroids and antibiotics.Oral steroids clinically meaningfully improved TDI for patients of all etiologies.	4

*PVOD, postviral olfactory dysfunction; CCCRC, connecticut chemosensory clinical research center test; BTT, butanol threshold test; CCSIT, cross-cultural smell identification Test; T&T, toyota & takagi olfactometer; TDI, threshold-discrimination-identification score; VAS, visual analog scale; BDP-spray, beclomethasone dipropionate spray*.

About the local steroids, nasal spray and injection to the olfactory cleft were included. Four studies evaluated the effect of nasal spray or injection alone on the olfaction in patients with PVOD (Heilmann et al., 2004; Fukazawa, 2005; Stenner et al., 2008; Seo et al., 2009; Fleiner and Goktas, 2011; Vaira et al., 2021). A retrospective study by Heilmann et al. reported the effect of mometasone spray at 0.1 mg twice daily for up to 3 months in patients with PVOD and there was no positive effect on olfactory dysfunction (Heilmann et al., 2004). Fukazawa et al. found injecting steroids into the nasal mucosa near the olfactory cleft significantly improved the T&T score and 49.6% of patients achieved the decrease in recognition threshold >1 point (Fukazawa, 2005). Furthermore, a prospective study by Fleiner and Goktas showed an increased efficacy of twice-daily beclomethasone injection to the olfactory cleft when compared with mometasone spray alone, with 25% of PVOD patients achieving an increase of more than six points in the TDI score (Fleiner and Goktas, 2011). Two studies used steroids in combination with other drugs (Seo et al., 2009; Vaira et al., 2021). Both the combination therapy (oral steroid and nasal steroid spray) and mixed therapy (oral steroid, nasal steroid spray, and *Ginkgo biloba*) significantly increased the olfaction in patients with PVOD, while there was no significant difference in the treatment response (defined as a score increase in BTT score ≥3) between these two treatment modalities (Seo et al., 2009). The novel therapy about systemic steroids and nasal irrigation with betamethasone, ambroxol, and prednisolone in patients with COVID-19 had been explored by Vaira et al. (2021). Compared with the no treatment group, the experimental group showed significant olfactory improvement in which the mean CCCRC score was from 10 to 60. Another retrospective case series trial by Stenner et al. investigated the use of neomycin spray to treat olfactory disorders including PVOD (34.8%) (Stenner et al., 2008). The administered regimen included beclomethasone at 3 mg daily for 20 days and then patients were randomly assigned to budesonide spray 1.5 mg twice daily vs. neomycin spray 7.5 mg twice daily in the control group for 12 weeks. After the experiment, there was no difference in recovery rate with additional antibiotics compared with topical treatment.

In summary, the routine use of nasal steroid spray alone during the management of PVOD without combination with other treatments is not recommended. However, direct injection of steroid or nasal steroid spray into the olfactory cleft has been proved to be a promising therapy, which needed further studies. Although early evidence suggests that systemic steroids are more effective than intranasal steroid spray in patients with olfactory loss due to varied etiologies (Kim et al., 2017), a study by Heilmann et al. suggested that there was no statistical difference between systemic steroids and intranasal steroid spray in treating patients with PVOD (Heilmann et al., 2004). More RCTs are required before a recommendation on medical treatment can be provided.

### Classical Olfactory Training

COT was firstly described by Hummel et al., which involved four odors including rose, eucalyptus, lemon, and cloves (Hummel et al., 2009). Patients usually smell each odorant for 10 s or longer twice daily until they have finished the entire set (Hummel et al., 2009). Five studies assessed the effect of COT in patients with PVOD (Table 2) (Hummel et al., 2009; Konstantinidis et al., 2013, 2016; Geißler et al., 2014; Gellrich et al., 2018).

**Table 2:** Summary of classical olfactory training studies included in the systematic review.

**References**	**Design**	**Patients**	**Intervention**	**Treatment details**	**The time of initiation of therapy**	**Follow-up**	**Improvement**	**Conclusion**	**Level of evidence**
Hummel et al. (2009)	Prospective controlled, non-blinded trial	COT (*n* = 24) vs. Control group (*n* = 11) PVOD: 35/56 Post-traumatic: 7/56 Idiopathic: 14/56	(1) Classical olfactory training (2) Control group	(1) Exposure to four odors twice daily(2) No treatment	4.3 years after clinical onset	12 weeks	An increase in Sniffin' Sticks score ≥6 COT: 20.8% Control: 0.9%	Patients including PVOD undergoing OT exhibited significantly higher scores than patients who did not train.	4
Gellrich et al. (2018)	Prospective controlled trial	COT (*n* = 30) vs. Control group (*n* = 31) All PVOD	(1) Classical olfactory training (2) Control group	(1) Exposure to four odors twice daily(2) No treatment	2.8 years after clinical onset	12 weeks	An increase in Sniffin' Sticks score ≥5.5 COT: 53.3% Control: no data COT group Threshold: 1.8 to 4 Discrimination: 7.3 to 9.5 Identification: 7.3 to 9 The mean TDI score: 16.4 to 21.9 (*p* < 0.01)	Even with the short olfactory duration, the points of Sniffin' Sticks could significantly enhance.	4
Konstantinidis et al. (2013)	Prospective controlled trial	COT (*n* = 49) vs. Control group (*n* = 32) PVOD: 81/119 Post-traumatic: 38/119	(1) Classical olfactory training (2) Control group	(1) Exposure to four odors twice daily(2) No treatment	10 months after clinical onset	16 weeks	An increase in Sniffin' Sticks score ≥6 Week 16 COT: 67.8% Control: 33% COT group Threshold: 2.3 to 2.6 Discrimination: 7.9 to 8.3 Identification: 8.8 to 9.6 The mean TDI score: 18.95 to 25.2 (*p* < 0.001) Control group Threshold: 2.4 to 2.5 Discrimination: 7.8 to 10.4 Identification: 8.6 to 12.2 The mean TDI score: 19 to 20.5 (*p* > 0.001)	Patients with training benefit increased mainly their identification and discrimination scores; a 16-week short-term exposure to specific odors may increase olfactory sensitivity in patients with PVOD.	4
Geißler et al. (2014)	Prospective, non-randomized case series	All PVOD (*n* = 39)	Classical olfactory training (all)	Exposure to four odors twice daily	10 months after clinical onset	16 and 32 weeks	An increase in Sniffin' Sticks score ≥6: PVOD Week 16: COT: 56% Week 32: COT: 79% Week 32 Threshold: 1 to 2 Discrimination: 8 to 10 Identification: 8 to 9 The mean TDI score: 17 to 21 (*p* = 0.021)	A longer duration of training could increase the effectiveness of training in comparison with a shorter training period.	4
Konstantinidis et al. (2016)	Prospective, randomized controlled trial	Long-term training group (*n* = 34) vs. Short-term training group (*n* = 36) vs. Control group (*n* = 41) All PVOD	(1) Classical olfactory training (2) Control group	Exposure to four odors twice daily(1) OT for 56 weeks(2) OT for 16 weeks(3) No treatment	9 months after clinical onset	56 weeks	An increase in Sniffin' Sticks score ≥6 Long: 71% Short: 58% Control: 37% Long-term group Threshold: 2.1 to 2.7 Discrimination: 5.3 to 10.2 Identification: 8.1 to 14.4 The mean TDI score: 15.9 to 27.3 (*p* < 0.05)	Short-term olfactory training results in sustainable olfactory improvement greater than that of the natural course of the disease.Long-term olfactory training presented an improvement of olfactory function with a first fast recovery period of 16 weeks and a second slower period of 40 weeks.	1B

*PVOD, postviral olfactory dysfunction; OT, olfactory training; COT, classic olfactory training; MOT, modified olfactory training*.

**Figure 2:**
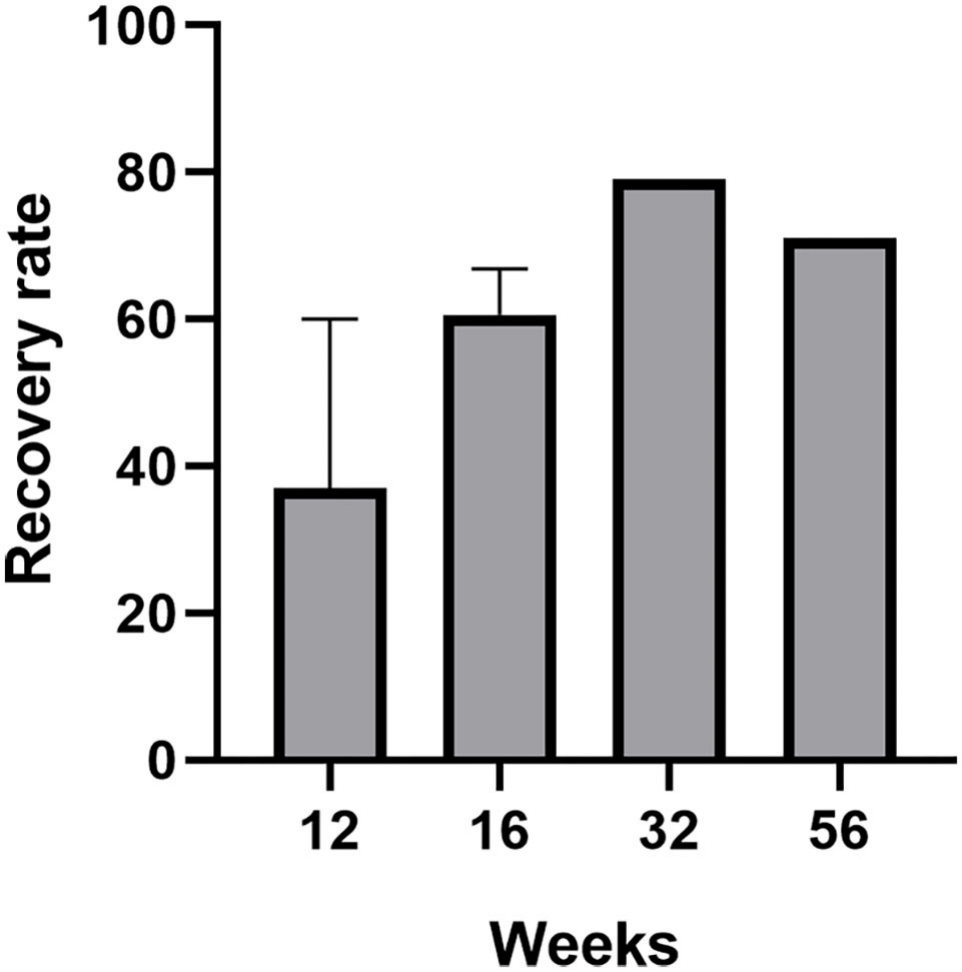
The recovery rate (defined as an increased ≥6 in TDI score) of patients with PVOD who used classical olfactory training with different treatment duration (12, 16, 32, and 56 weeks).

Four of these trials compared COT to placebo with the follow-up varied from 8 to 56 weeks, and all the studies proved the efficiency of the COT on patients with PVOD (Hummel et al., 2009; Konstantinidis et al., 2013, 2016; Gellrich et al., 2018). Even for a short period of 12 weeks, the COT group provided relatively higher scores than to placebo which did not receive training (Hummel et al., 2009; Gellrich et al., 2018). The percentage of patients achieving MCID (defined as a ≥6 increase in TDI scores) in the COT and control group was 20.8 and 0.9%, respectively (Hummel et al., 2009). Another study by Konstantinidis et al. showed that patients achieving MCID in COT and control group were 67.8 and 33%, respectively (Konstantinidis et al., 2013). The mean TDI scores significantly changed from 18.95 to 25.2 and patients with training benefit increased mainly their identification and discrimination scores (discrimination: 7.9 to 8.3, identification: 8.8 to 9.6) (Konstantinidis et al., 2013). Furthermore, another prospective and non-randomized study without control by Geißler et al. showed that the percentage of patients with PVOD achieving MCID was significantly higher after 32 weeks of olfactory training (79%) than that after 16 weeks of olfactory training (56%) (Geißler et al., 2014). A prospective, randomized controlled clinical trial by Konstantinidis et al. investigated different olfactory training duration in patients with PVOD (Konstantinidis et al., 2016). Patients with PVOD were exposed to four odors twice daily for 16 weeks (short-term group) or 56 weeks (long-term group) and compared with the no treatment group. Treatment response was defined as an increase of TDI scores ≥6. This study found that the treatment response rates in the long-term group, short-term group, and control group were 71, 58, and 37%, respectively. Besides, the long-term COT group had a significant increase in TDI score changing from 15.9 to 27.3. The MCID in COT showed a high rate with the increase of treatment time, reaching 79% in 32 weeks, but there had a tendency to decrease beyond 32 weeks which was 71% in 56 weeks (Figure 2).

In summary, short-term olfactory training results in sustainable olfactory improvement greater than that of the natural course of the disease. Given the positive results from multiple clinical studies, a recommendation is designated for long-term use (>12 weeks) for olfactory training in patients with PVOD. Furthermore, olfactory function sustained a fast recovery period and a second slower period after long-term olfactory training.

### Modified Olfactory Training

Four randomized controlled and one pseudorandomized study used modifications of the COT, including different concentrations of odorants, different molecular odorants, various odorants, and olfactory balls (Table 3) (Damm et al., 2014; Altundag et al., 2015; Poletti et al., 2017; Qiao et al., 2020; Saatci et al., 2020). Two RCTs without a placebo group compared two different types of olfactory training with COT for weeks and all the treatment groups showed significant olfactory improvement after olfactory training (Qiao et al., 2020; Saatci et al., 2020). A study by Qiao et al. compared four different odors with COT twice daily for 6 months, and there was no significant difference in TDI improvements between the two groups (Qiao et al., 2020). Another study by Saatci et al. utilized an olfactory training ball for 12 weeks and patients receiving olfactory training ball exhibited significantly higher TDI scores than patients treated with COT (Saatci et al., 2020).

**Table 3:** Summary of modified olfactory training studies included in the systematic review.

**References**	**Design**	**Patients**	**Intervention**	**Treatment details**	**The time of initiation of therapy**	**Follow-up**	**Improvement**	**Conclusion**	**Level of evidence**
Qiao et al. (2020)	Prospective, randomized non-blinded controlled trial	Control group (*n* = 60) vs. Test group (*n* = 65) All PVOD	(1) Classical olfactory training (2) Training with four different odors	(1) Exposure to four odors twice daily(2) Exposure to four different odors twice daily	1 year after clinical onset	24 weeks	An increase in Sniffin' Sticks score ≥6 Control: 41.54% Test: 41.67% Control group Threshold: 6.8 to 6.9 Discrimination: 7.2 to 9.5 Identification: 2.9 to 6.1 The mean TDI score: 16.8 to 22.5 (*p* < 0.05) Test group Threshold: 6.5 to 6.6 Discrimination: 7.1 to 9.7 Identification: 2.8 to 6.4 The mean TDI score: 16.3 to 22.9 (*p* < 0.05)	In patients with PVOD, there was no significant difference in Sniffin' Sticks score improvements between combinations.The prolonged and earlier start of olfactory training would be helpful for the recovery of olfactory functions.	2B
Saatci et al. (2020)	Prospective, randomized non-blinded controlled trial	COT (*n* = 60) vs. OTB (*n* = 60) All PVOD	(1) Classical olfactory training (2) Olfactory training ball	(1) Exposure to four odors twice daily(2) Exposure to four odors in a sphere-shaped ball twice daily	10 years after clinical onset	12 weeks	An increase in Sniffin' Sticks score ≥5.5 COT: 30% OTB: 70% COT group Threshold: 2.8 to 2.9 Discrimination: 6.5 to 7.6 Identification: 7.0 to 9.3 The mean TDI score: 16.2 to 19.9 (*p* < 0.001) OTB group Threshold: 2.7 to 3.1 Discrimination: 6.6 to 9.1 Identification: 6.7 to 9.8 The mean TDI score: 16.1 to 22.1 (*p* < 0.001)	Patients undergoing OTB exhibited significantly higher scores than patients who were treated with OT.	2B
Altundag et al. (2015)	Prospective, randomized, controlled clinical trial	MOT (*n* = 37) vs. COT (*n* = 33) vs. Control group (*n* = 15) All PVOD	(1) Modified olfactory training (2) Classical olfactory training (3) Control group	(1) Exposure to four odors twice daily for 36 weeks(2) Exposure to four odors twice daily for 12 weeks, followed by four different odors for 12 weeks, followed by four different odors for 12 weeks(3) No treatment	7 months after clinical onset	12, 24, and 36 weeks	An increase in Sniffin' Sticks score ≥6 Week 36 MOT: 56% COT: 46% MOT group Threshold: 2.4 to 2.8 Discrimination: 7.7 to 10.9 Identification: 8 to 12.6 The mean TDI score: 18.1 to 26.3 (*p* < 0.001) COT group Threshold: 2.5 to 2.7 Discrimination: 7.5 to 10.1 Identification: 8.2 to 11.5 The mean TDI score: 18.2 to 24.3 (*p* < 0.001) Control group Threshold: 2.5 to 2.6 Discrimination: 7.4 to 8.1 Identification: 8.1 to 8.9 The mean TDI score: 18 to 19.7 (*p* ≤ 0.05)	Changing the types of odors periodically during OT can enhance the likelihood of success of this olfactory therapy.	1B
Poletti et al. (2017)	Prospective, pseudorandomized trial	Light molecular weight OT (*n* = 37) vs. Heavy molecular weight OT (*n* = 33) PVOD: 70/96 Post-traumatic: 26/96	(1) Training with light molecular weight odorants (2) Training with heavy molecular weight odorants	Exposure to three odors twice in the morning and twice in the evening(1) Light molecular weight odorants <150 g/mol(2) Heavy molecular weight odorants >150 g/mol	–	20 weeks	An increase in Sniffin' Sticks score ≥5.5 All PVOD: 45% Light molecular group Threshold: 2.8 to 3.9 Discrimination: 8.5 to 10.5 Identification: 7.4 to 8 The mean TDI score: 18.6 to 22.3 (*p* = 0.021) Heavy molecular group Threshold: 1.9 to 4.5 Discrimination: 9.1 to 11.1 Identification: 6.9 to 7.5 The mean TDI score: 17.7 to 23 (*p* < 0.001)	In patients with PVOD, training with heavy-weight molecules produced an improved threshold compared with light-weight molecules; except for threshold scores, there were no differences between LWM and HWM.	4
Damm et al. (2014)	Randomized, single-blind, controlled crossover clinical trial	High-training group (*n* = 70) vs. Low-training group (*n* = 74) All PVOD	(1) Training with high concentration odorants (2) Training with low concentration odorants (3) Crossover in treatment regimen at 18 weeks	(1) Exposure to four odors twice daily(2) Exposure to four odors twice daily of concentration at the 10th percentile of the threshold of healthy volunteers	10.5 months after clinical onset	18 and 36 weeks	An increase in Sniffin' Sticks score ≥6 Week 36: High: 45.8% Low: 30.8% Low–high training group Threshold: 2.6 to 3.42 Discrimination: 8 to 10 Identification: 7.54 to 8.7 The mean TDI score: 18.2 to 22.1 (*p* < 0.001) High–low training group Threshold: 2.8 to 3.5 Discrimination: 7.9 to 9.9 Identification: 7.15 to 8.9 The mean TDI score: 17.9 to 22.3 (*p* < 0.001)	The use of odors at higher concentrations is beneficial to PVOD; it seems particularly useful in patients who start OT within 12 months after the onset of the disorder (*p* = 0.03).	2B

*PVOD, postviral olfactory dysfunction; OT, olfactory training; COT, classic olfactory training; MOT, modified olfactory training; LWM, light molecular weight odorants; HWM, heavy molecular weight odorants; OTB, olfactory training ball*.

Another prospective, randomized, controlled clinical trial by Altundag et al. compared the effect of modified olfactory training (three sets of four different odors sequentially), COT, and no intervention in patients with PVOD (Altundag et al., 2015). Both the modified olfactory training and COT groups reached better scores than controls in terms of odor discrimination and odor identification. Besides, changing the types of odors periodically during olfactory training can enhance the likelihood of success of this olfactory therapy.

**Figure 3:**
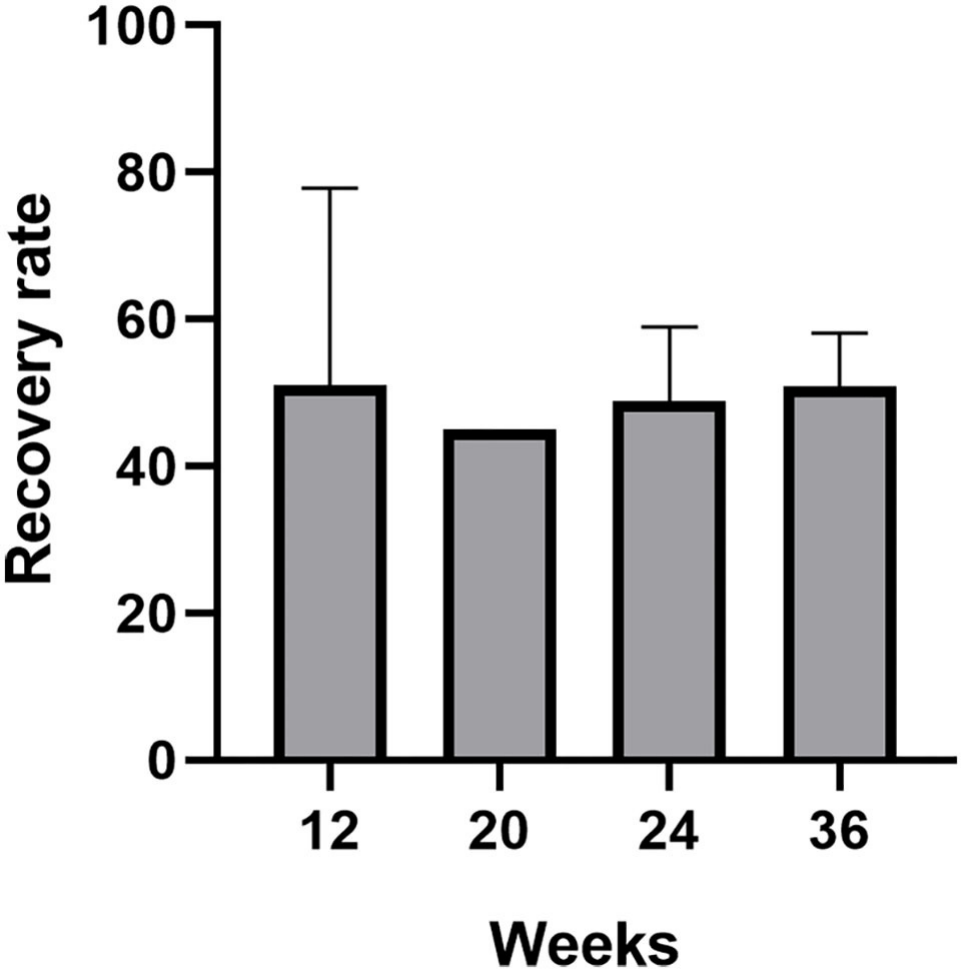
The recovery rate (defined as an increased ≥6 in TDI score) of patients with PVOD who used modified olfactory training with different treatment duration (12, 20, 24, and 36 weeks).

Another two prospective studies explored the effect of different odor concentrations and molecular weight odorants on the olfactory function in patients with PVOD (Damm et al., 2014; Poletti et al., 2017). An RCT evaluated heavy molecular weight odorants, followed by more than 150 g/mol four times per day compared with light molecular weight odorants (Poletti et al., 2017). Heavy-weight molecules were associated with a larger improvement in threshold score at 5 months. Except for the threshold, there were no differences between light molecular weight odorants and heavy molecular weight odorants. Damm et al. performed different concentrations of odorants treating patients in PVOD and follow-up was 18 and 36 weeks (Damm et al., 2014). The use of odors at higher concentrations was more beneficial to PVOD than low concentrations of odorants within 12 months. At 18 weeks, 25.7% of the high-training group enhanced their olfactory function and achieved MCID. However, only 14.9% of the low-training group reported MCID. In either the high (45.8%) or the low group (30.8%), more patients had improvement at 36 weeks. The rate of olfactory recovery (defined as an increased ≥6 in TDI score) in all studies referred to modified olfactory training increased separately around 50% (51, 25.7, 45, 48.8, 50.9%) (Figure 3). There was no significant difference in the recovery rate with prolonged treatment.

In summary, a recommendation supports the use of modified olfactory training for patients with PVOD. On the other hand, they are more helpful for the recovery of olfactory dysfunction on an earlier and prolonged start.

### Combination of Classic Olfactory Training and Steroid Treatment

Five trials studied combined therapy of olfactory training and steroid vs. the control group (Table 4) (Fleiner et al., 2012; Nguyen and Patel, 2018; Abdelalim et al., 2021; Kasiri et al., 2021; Le Bon et al., 2021). The control group used COT only and the experimental group used COT and steroids. The treatment details involved exposure to four odors twice daily and budesonide 0.5 mg twice daily for 4 or 8 months; 57.1% of patients receiving a combination of COT and topical steroids achieved an increase in TDI score ≥6 and a combination of COT and topical steroids could enhance the efficacy of COT (Fleiner et al., 2012). Another study by Nguyen and Patel examined the effect of adding budesonide irrigation to olfactory training on patients with olfactory loss (Nguyen and Patel, 2018); 42.6% of patients with PVOD had a clinically significant change (defined as a ≥5 increase in UPSIT scores). Abdelalim and Kasiri investigated the efficiency of mometasone nasal spray combination with COT in COVID-19 patients (Abdelalim et al., 2021; Kasiri et al., 2021). The recovery of olfactory dysfunction in the control group was 62% higher than 52% in the COT and nasal steroids group. One RCT evaluated the efficacy of COT and oral corticosteroids in patients with olfactory dysfunction secondary to COVID-19 (Le Bon et al., 2021). Patients in the COT and steroids group had improved their olfactory score by 7.7 points on average (*p* = 0.007), compared with a 2.1-point increase in the COT group (*p* = 0.126).

**Table 4:** Summary of combination with olfactory training and steroid studies included in the systematic review.

**References**	**Design**	**Patients**	**Intervention**	**Treatment details**	**The time of initiation of therapy**	**Follow-up**	**Improvement**	**Conclusion**	**Level of evidence**
Fleiner et al. (2012)	Retrospective case series	COT (*n* = 9) vs. COT + steroids group (*n* = 7) PVOD: 16/46 Post-traumatic: 7/46 Sinonasal: 15/46 Idiopathic: 8/46	(1) Classic olfactory training only (2) Classic olfactory training + topical corticosteroids	(1) Exposure to four odors twicedaily(2) Topical corticosteroid treatment not specified	21 months after clinical onset	16 and 32 weeks	An increase in Sniffin' Sticks score ≥6 Month 4 COT: 11.1% COT + steroids: 14.3% Month 8 COT: 11.1% COT + steroids: 57.1%	In all PVOD patients, the improvement of Sniffin' Sticks score caused by COT exhibited little clinical significance; a combination of COT and steroids could enhance the efficacy of COT	4
Nguyen and Patel (2018)	Randomized controlled trial	Control group (*n* = 30) vs. Budesonide irrigation group (*n* = 32) PVOD: 62/133 Idiopathic: 46/133 Medication-related: 6/133 Post-traumatic: 16/133 Environmental exposure: 3/133	(1) Classic olfactory training + saline irrigation (2) Classic olfactory training + budesonide irrigation	(1) Exposure to four odors twice daily and saline irrigations twice daily(2) Exposure to four odors twice daily and budesonide 0.5 mg twice daily	1–2 years after clinical onset	24 weeks	An increase in UPSIT score ≥5 All patients Control group: 26.9% Budesonide irrigation group: 43.9% All PVOD: 42.6%	Olfactory training with budesonide irrigation significantly improves olfaction compared with olfactory training using saline irrigation alone	1B
Abdelalim et al. (2021)	Prospective, randomized controlled trial	COT (*n* = 50) vs. COT + steroids group (*n* = 50) All COVID-19	(1) Classic olfactory training only (2) Classic olfactory training + mometasone nasal spray	(1) Exposure to four odors twicedaily(2) Exposure to four odors twice daily and mometasone two puffs/nasal cavity once daily	12 days after clinical onset	3 weeks	VAS score improved to 10 COT: 62% COT + steroids: 52%	This topical corticosteroid nasal spray shows no superiority in the treatment of post-COVID-19 anosmia over the olfactory training	1B
Kasiri et al. (2021)	Prospective, randomized controlled trial	COT + placebo group (*n* = 38) vs. COT + steroids group (*n* = 39) All COVID-19	(1) Classic olfactory training + saline spray (2) Classic olfactory training + mometasone nasal spray	(1) Exposure to four odors twiceDaily and saline two puffs/nasal cavity twice daily(2) Exposure to four odors twice daily and mometasone two puffs/nasal cavity twice daily	2 weeks after clinical onset	4 weeks	The changes in VAS score, mean (SD), the rate of normal smell after therapy COT + placebo: 5.7 (1.6), 48.7% COT + steroids: 5.2 (2.3), 21.1% (*p* = 0.329)	Compared with olfactory training, mometasone furoate nasal spray combination with olfactory training showed a higher improvement in severe chronic anosmia by COVID-19	1B
Le Bon et al. (2021)	Prospective, pseudorandomized trial	COT (*n* = 18) vs. COT + OCS group (*n* = 9) All COVID-19	(1) Classic olfactory training only (2) Classic olfactory training + oral corticosteroids	(1) Exposure to four odors twice daily(2) Exposure to four odors twice daily and a 10-day course of 32 mg of methylprednisolone once daily	5 weeks after clinical onset	10 weeks	Sniffin' Sticks Patients in the OCS + COT group had improved their olfactory score by 7.7 points on average (*p* = 0.007), compared with a 2.1-point increase in the COT group (*p* = 0.126)	This study supports a combination of oral corticosteroids and olfactory training is more efficient than olfactory training only in the management of olfactory dysfunction resulting from COVID-19 infection	4

*PVOD, postviral olfactory dysfunction; OT, olfactory training; COT, classic olfactory training; UPSIT, the university of Pennsylvania smell identification Test; VAS, visual analog scale; OCS, oral corticosteroids group*.

In summary, olfactory training with steroids exhibits clinical significance in the improvement of the Sniffin' Sticks score. Although some studies showed there were no differences between a combination of nasal spray and olfactory training and olfactory training only, the use of steroid irrigation and oral corticosteroids in addition to COT could be beneficial in accelerating the recovery of PVOD. A combination of steroids and olfactory training is more efficient than olfactory training only in managing olfactory dysfunction from PVOD.

## Discussion

This is the first systematic review summarizing the evidence for steroids, olfactory training, or both interventions in the treatment of PVOD and revealed the improvement and optimization of treatment modalities. For steroid treatment, direct injection of steroid or nasal steroid spray into the olfactory cleft significantly improved the olfactory function in patients with PVOD and nasal steroid spray alone is not recommended (Heilmann et al., 2004; Fukazawa, 2005; Fleiner and Goktas, 2011). These studies indicated that direct alleviation of the lesions in the olfactory cleft facilitated the olfactory improvement in patients with PVOD. It has been reported that nasal steroid sprays deliver medication to the restricted areas including the anterior and inferior parts of the nasal cavity and the deposition of steroids in the superior and posterior of the nasal passages is relatively limited (Djupesland and Skretting, 2012; Lam et al., 2013; Emanuel et al., 2014). That might explain why local steroids direct into the olfactory cleft demonstrated exact treatment effect in patients with PVOD. Similarly, head positions including Kaiteki and Mygind positions delivered the nasal drops effectively to the olfactory epithelium, and further studies should evaluate the effect of nasal steroid drops with these two head positions in patients with PVOD (Mori et al., 2016; Milk et al., 2021). When comparing the uses of steroids in patients with PVOD, two studies showed the TDI scores improved from 14.39 to 18.86, 15.2 to 16.9 in the systemic steroids group, while the TDI scores even decreased in the nasal steroids group (Jaeschke et al., 1989; Schriever et al., 2012). It should be pointed out that current evidence supporting systemic steroids over nasal steroids spray in patients with PVOD is limited and more studies are needed (Heilmann et al., 2004; Schriever et al., 2012).

We next systematically reviewed the efficiency of different olfactory training modalities in patients with PVOD from the perspective of olfactory recovery rate. Recent meta-analyses found a beneficial effect from olfactory training on a range of etiologies including PVOD for olfactory dysfunction, although characterized by a high level of heterogeneity among included studies (Pekala et al., 2016). A consensus about the treatment of PVOD suggested that olfactory training was an overwhelming recommendation for olfactory therapy in patients with PVOD (Pekala et al., 2016; Sorokowska et al., 2017; Kattar et al., 2020; Addison et al., 2021). Although there is growing evidence supporting the efficiency of COT in patients with PVOD, the exact effect of modification of the COT including treatment duration, various odorants, olfactory training device (olfactory training ball), changing the types of odors periodically, different molecular odorants, and different concentrations of odorants on patients with PVOD is not systematically reviewed.

This systematic review firstly showed that more patients with PVOD would achieve MCID after long-term COT (16 weeks, 60.6%; 32 weeks, 79%; 56 weeks, 71%) than those after short-term COT (12 weeks, 37.05%) (Figure 2) (Hummel et al., 2009; Konstantinidis et al., 2013, 2016; Geißler et al., 2014; Gellrich et al., 2018). It can be inferred that long-term olfactory training provided sustainable improvement of at least 56 weeks and the training effect consistently modulated the olfactory system. Recent fMRI studies on patients with PVOD showed that olfactory training reorganized functional connectivity networks, especially within the visual cortex (Kollndorfer et al., 2014; Jiramongkolchai et al., 2021). An extended period of odor exposure maintained the olfactory training effect at a sustainable high level. However, the mechanism accounting for the difference between short-term and long-term effects is still unknown.

As for the types of odorant, MOT with four different odors (essential balm, vinegar, alcohol, and rose perfume) was not superior to COT regarding the difference in TDI improvements (Qiao et al., 2020). This study demonstrated that different types of olfactory agents which irritated the olfactory system and nasal trigeminal system functioned similarly in improving olfaction. A new olfactory training device called olfactory training ball increased adherence to the training process, which was associated with better olfactory outcomes than COT (Saatci et al., 2020). Interestingly, changing the odors with prolonged olfactory training duration (>12 weeks) would enhance the likelihood of success of this therapy (Altundag et al., 2015). Furthermore, continuing olfactory training with four different odors after 12 and 24 weeks produced better results in terms of odor discrimination and odor identification scores as compared with COT throughout the entire study. Previous studies showed that it was odor discrimination and odor identification and not odor thresholds that correlated significantly with tests of executive function and semantic memory which is highly associated with central processing and cognitive function (Nasreddine et al., 2005; Hedner et al., 2010). We speculated that changing the odors with prolonged olfactory training produced cognitive improvement, which further leads to improved olfactory perception.

As for the different molecular weight odorant and odor concentrations, heavy-weight molecules were associated with a larger improvement in threshold score, and more patients with PVOD would achieve MCID in the high-concentration training group than that in the low-concentration training group (Damm et al., 2014; Poletti et al., 2017). A recent meta-analysis showed that it was the odor discrimination and odor identification but not odor thresholds that improved after olfactory training among patients with olfactory loss due to varied etiologies (Pekala et al., 2016). It seems that changes in molecular weight odorant during olfactory training would facilitate the improvement of odor thresholds, which provided a new strategy to comprehensively improve olfactory function in patients with PVOD.

We also summarized the olfactory recovery rates (defined as an increased ≥6 in TDI score) in patients with different MOT and also found that the recovery rate (50% on average) did not significantly change among prolonged treatment duration (at weeks 12, 20, 24, and 36) (Figure 3) (Damm et al., 2014; Altundag et al., 2015; Poletti et al., 2017; Qiao et al., 2020; Saatci et al., 2020). Currently, studies with a treatment duration of MOT >36 weeks in patients with PVOD are still lacking. Based on the above evidence, we speculated that the modification of COT did not change the percentage of patients achieving MCID. Moreover, further studies of direct comparison between various MOT and COT with treatment duration >36 weeks are warranted.

A thorough discussion on whether a combination of steroids and olfactory training is better than monotherapy has not been reported. It is imperative to confirm the efficacy of steroids and olfactory training on patients with PVOD. Fleiner et al. showed 57.1% of patients receiving a combination of COT and topical steroids achieved an increase in TDI score ≥6 and a combination of COT and topical steroids could enhance the efficacy of COT (Fleiner et al., 2012). Similarly, an RCT by Nguyen and Patel showed that using budesonide irrigation with COT was superior to COT alone and 42.6% of patients with PVOD had a clinically significant change (defined as a ≥5 increase in UPSIT scores) (Nguyen and Patel, 2018). It can be inferred that the addition of steroids including nasal steroid spray or steroid irrigation to COT could significantly improve the efficiency of COT within 8 months. Presumably, local steroids could suppress inflammation within sinonasal cavities that caused anosmia and promote proper neuronal regeneration at the same time to enhance the effect of COT (Nguyen and Patel, 2018). During the COVID-19 pandemic, exploring the treatment of patients with olfactory dysfunction was important. This systematic review also compared combined olfactory training and steroids with olfactory training alone in patients with post-COVID-19 anosmia. Consensus guidelines had identified the appropriateness of olfactory training for all patients with olfactory dysfunction of more than 2 weeks duration, and topical and systemic steroids may be considered based on the use of olfactory training (Hopkins et al., 2021). Two RCTs showed olfactory improvement in treatment with a combination of nasal spray and olfactory training (Abdelalim et al., 2021; Kasiri et al., 2021). Furthermore, a higher improvement in severe chronic anosmia by COVID-19 was observed. Interestingly, another study by Le et al. showed that a combination of COT and oral steroids for 10 weeks was more effective than olfactory training only in patients with COVID-19 (Le Bon et al., 2021). However, considering the systemic side effects, it is not recommended to use oral corticosteroids more than 2 weeks with persistent olfactory dysfunction after COVID-19 (Hopkins et al., 2021). Given the available evidence, large randomized controlled trials are needed to verify the exact effect of steroids with COT in patients with PVOD.

It should be pointed out that there was a strong association between the time of initiation of therapy and the recovery rate after treatment in patients with PVOD, with more improvement the earlier the initiation of therapy including steroids and olfactory training. It can be inferred that patients would benefit more when the time of initiation of therapy starts earlier. It has been proposed that steroids potentiated the effects of olfactory training by dampening any inflammation that could be causing or exacerbating olfactory loss (Nguyen and Patel, 2018).

The limitations of this study include a small number of randomized controlled trials and a lack of enough control groups. The lack of sufficient study and quantifiable data in the meta-analysis limits results and evidence. Some studies used no interventions and other used placebos for the control group. RCTs are needed to verify the effect of steroid, olfactory training, or a combination of both in patients with PVOD.

## Conclusion

With the accumulation of studies exploring the effects of steroids or olfactory training in patients with PVOD, evidence focused on the improvement and optimization of these treatment modalities is expanding. Direct injection of steroid or nasal spray into the olfactory cleft proved to be a promising therapy for patients with PVOD. Patients with PVOD would benefit more from long-term COT (>12 weeks). Factors including treatment duration, various odorants, olfactory training devices, changing the types of odors periodically, different molecular odorants, and different concentrations of odorants tended to increase the efficiency of MOT. Furthermore, a combination of COT and topical steroids can significantly improve olfactory function in patients with PVOD. More additional randomized controlled trials related to combined steroids irrigation or spray with COT are needed.

## Data Availability Statement

The original contributions presented in the study are included in the article/supplementary material, further inquiries can be directed to the corresponding author/s.

## Author Contributions

All authors have made substantial contributions to the conception, analysis, and interpretation of data in this article, approved the submitted version, and agreed to be personally accountable for our contributions and to ensure that questions related to the accuracy or integrity of any part of the work, even ones in which we are not personally involved, are appropriately investigated and resolved, and the resolution documented in the literature.

## Conflict of Interest

The authors declare that the research was conducted in the absence of any commercial or financial relationships that could be construed as a potential conflict of interest.

## Publisher's Note

All claims expressed in this article are solely those of the authors and do not necessarily represent those of their affiliated organizations, or those of the publisher, the editors and the reviewers. Any product that may be evaluated in this article, or claim that may be made by its manufacturer, is not guaranteed or endorsed by the publisher.

## Abbreviations

PVOD: postviral olfactory dysfunction
URTI: upper respiratory tract infection
COVID-19: novel coronavirus disease 2019
SARS-CoV-2: severe acute respiratory syndrome coronavirus 2
PRISMA: preferred reporting items for systematic reviews and meta-analyses
BTT: butanol threshold testing
CCSIT: cross-cultural smell identification test
CCCRC: connecticut chemosensory clinical research center test
T&T: toyota & takagi olfactometer
UPSIT: university of pennsylvania smell identification test
PEA: phenyl ethyl alcohol threshold testing
VAS: visual analog scale
COT: classical olfactory training
MOT: modified olfactory training
MCID: minimal clinically important difference.
